# Serum irisin levels are decreased in patients with sepsis, and exogenous irisin suppresses ferroptosis in the liver of septic mice

**DOI:** 10.1002/ctm2.173

**Published:** 2020-09-29

**Authors:** Shasha Wei, Jianbin Bi, Lifei Yang, Jia Zhang, Yafeng Wan, Xue Chen, Yawen Wang, Zheng Wu, Yi Lv, Rongqian Wu

**Affiliations:** ^1^ National Local Joint Engineering Research Center for Precision Surgery & Regenerative Medicine Shaanxi Provincial Center for Regenerative Medicine and Surgical Engineering First Affiliated Hospital of Xi'an Jiaotong University Xi'an Shaanxi Province China; ^2^ Department of Hepatobiliary Surgery First Affiliated Hospital of Xi'an Jiaotong University Xi'an Shaanxi Province China; ^3^ BioBank, First Affiliated Hospital of Xi'an Jiaotong University Xi'an Shaanxi Province China

**Keywords:** ferroptosis, GPX4, irisin, mitochondrial function, sepsis

## Abstract

**Background:**

Sepsis remains a major health issue without an effective therapy. Ferroptosis, an iron‐dependent programmed cell death, has been proposed to be related to the pathogenesis of sepsis. Irisin, a myokine released during exercise, improves mitochondrial function under various conditions. Ferroptosis is closely related to mitochondrial function. However, the role of irisin in sepsis‐induced ferroptosis and mitochondrial dysfunction in the liver remained unknown. Thus, we hypothesize that irisin treatment suppresses ferroptosis and improves mitochondrial function in sepsis.

**Methods:**

To study this, we first explored the role of serum irisin levels in patients with sepsis, and then determined the effect of irisin administration on ferroptosis and mitochondrial function in the liver of septic mice.

**Results:**

Serum irisin levels were decreased and negatively correlated with the APACHE II scores in patients with sepsis. In mice subjected to cecal ligation and puncture (CLP), exogenous irisin administration suppressed ferroptosis, inhibited inflammatory response, decreased reactive oxygen species (ROS) production, restored abnormal mitochondrial morphology, and increased mtDNA copy number and adenosine triphosphate (ATP) content. The effect of irisin on ferroptosis was confirmed in LPS‐treated hepatocytes and CLP‐induced septic mice. Inhibition of glutathione peroxidase 4 (GPX4), a central regulator of ferroptosis, reduced irisin's protective effects in LPS‐treated hepatocytes and CLP‐induced septic mice, while blocking the irisin receptor with RGD peptide or Echistain decreased irisin‐induced GPX4 expression.

**Conclusions:**

Serum irisin levels are decreased and negatively correlated with disease severity in patients with sepsis, and irisin treatment suppresses ferroptosis and restores mitochondrial function in experimental sepsis. Irisin may offer therapeutic potential in the management of sepsis.

## Graphical abstract

Serum irisin levels are decreased and negatively correlated with disease severity in patients with sepsis. Irisin treatment suppresses ferroptosis and restores mitochondrial function in experimental sepsis. Irisin may provide therapeutic potential in the management of sepsis.

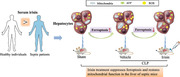

**Abbreviations**
ALT: alanine aminotransferase
APACHE: Acute Physiology and Chronic Health Evaluation
AST: aspartate aminotransferase
ATP: adenosine triphosphate
ATPB: energy generation related protein
CLP: cecal ligation and puncture
DHE: dihydroethidium
ELISA: enzyme‐linked immunosorbent assays
GPX4: glutathione peroxidase 4.
HMGB1: high mobility group box 1
LC3: microtubule associated protein 1 light chain 3
mtDNA: mitochondrial DNA
MT‐ND1: mitochondrial‐encoded NADH dehydrogenase 1
MTR: MitoTracker Red CMXRos
nDNA: nuclear DNA
OCT4: nuclear‐encoded internal control of POU class 5 homeobox 1
OMM: outer mitochondrial membrane
OXS: oxidative stress
qPCR: quantitative polymerase chain reaction
ROS: reactive oxygen species
SOFA: Sequential Organ Failure Assessment
TEM: transmission electron microscopy
TFAM: mitochondrial biogenesis related protein

## 1 BACKGROUND

Sepsis is a leading cause of in‐hospital deaths and has high morbidity.^1^ Although the introduction of modern intensive care has greatly improved survival rates, there are no specific drugs for sepsis.^2^ The liver plays a central role in the pathogenesis of sepsis.^3^, ^4^ Several studies have revealed that liver dysfunction occurs early in the development of sepsis.^5^, ^6^ Liver dysfunction is closely linked to the mortality rate in sepsis and early liver dysfunction is an independent risk factor for poor prognosis.^7^, ^8^, ^9^ Thus, protection against liver is very important for the treatment of sepsis. Mitochondria have multiple metabolic and signaling functions, such as ATP production, intracellular calcium homeostasis, ROS production, and apoptosis. Mitochondrial dysfunction closely related to sepsis‐induced organ dysfunction.^10^, ^11^ Clinical studies suggest that treatments focusing on mitochondrial dysfunction may improve the outcomes of patients with sepsis.^12^

Irisin, a peptide hormone cleaved from fibronectin type III domain‐containing 5 (FNDC5), was an exercise‐induced myokine promoting the browning of white adipose tissue.^13^ Irisin participates in hepatic glucose and lipid metabolism and that it also reduces oxidative stresses in hepatocytes.^14^, ^15^ Our group found that irisin has beneficial effects on mitochondrial biogenesis, mitochondrial function, and oxidative stress.^16^, ^17^, ^18^, ^19^

Ferroptosis discovered by Stockwell in 2012 is iron‐dependent and distinct from other types of programmed cell death.^20^ Increasing iron concentrations and ROS levels result in the occurrence of ferroptosis.^21^ Studies have shown that ferroptosis was involved in the pathological process of various diseases.^22^, ^23^, ^24^, ^25^ Recently, it was reported that ferroptosis modulated by GPX4 might be a novel pathophysiological mechanism of sepsis.^26^ Inhibition of ferroptosis exerts protective effects in sepsis‐induced heart injury.^27^ However, there is no research either on the role of ferroptosis in sepsis‐induced liver injury or on the association between irisin and ferroptosis in sepsis. Ferroptosis is closely related to mitochondrial function.^28^ However, the role of irisin in sepsis‐induced ferroptosis and mitochondrial dysfunction in the liver remained unknown. Thus, we hypothesize that irisin treatment suppresses ferroptosis and improves mitochondrial function in sepsis. We first explored the role of serum irisin levels in patients with sepsis, and then determined the effect of irisin administration on ferroptosis and mitochondrial function in the liver of septic mice.

## 2 METHODS

### 2.1 Patients

This observational study included 60 patients with sepsis (age  >  18 years) in the First Affiliated Hospital of Xi'an Jiaotong University between September 2016 and December 2018. Sepsis was diagnosed based on the results of blood culture and organ dysfunction according to the Sepsis‐3 criteria ((1) suspected infection and^2^ Sequential Organ Failure Assessment (SOFA) score ≥ 2), while patients with malignant tumors were excluded. Basic clinical data of the patients are presented in Table 1. The APACHE II score^29^ was used to evaluate the severity of sepsis. The blood samples were collected from septic patients within 24 h of hospital admission. The study was approved by the Ethics Committee of the First Affiliated Hospital of Xi'an Jiaotong University and all study participants signed the informed consent.

**TABLE 1:** Demographic data, comorbidities, and laboratory results of septic patients

Variable	Total (N = 60)	Irisin ≥ 12.67 ng/mL, N = 30	Irisin < 12.67 ng/mL, N = 30	*P*‐value
Age (years; mean ± SD)	59.3 ± 15.8	59.2 ± 14.4	59.4 ± 17.4	0.974
Gender (male/female)	34/26	14/16	20/10	0.118
Comorbidities (n, %)				
Hypertension	16 (26.6)	6 (20)	10 (33.3)	0.243
Diabetes mellitus	15 (25)	6 (20)	9 (30)	0.371
Virus hepatitis	10 (16.7)	2 (6.7)	8 (26.7)	**0.038**
Cirrhosis	7 (11.7)	1 (3.3)	6 (20)	**0.044**
Infectious source (n, %)				
Lung	12 (20)	4 (13.3)	8 (26.6)	0.197
Intraabdominal	22 (36.7)	13 (43.3)	9 (30)	0.284
Urinary	5 (8.3)	2 (6.7)	3 (10)	0.640
Skin	2 (3.3)	2 (6.7)	0 (0)	0.150
Other	19 (31.6)	9 (30)	10 (33.3)	0.781
Laboratory results (mean ± SD or median, interquartile range)				
Leucocytes (×10^9^/L)	8.8 ± 4.1	9.1 ± 4.8	8.5 ± 3.6	0.575
Platelet count (×10^9^/L)	147.9 ± 91.5	152.3 ± 101.2	143.6 ± 82.2	0.717
Neutrophils (×10^9^/L)	6.9 (4.0‐9.5)	6.9 (3.9‐10.2)	6.9 (4.3‐9.1)	0.582
Lymphocytes (×10^9^/L)	0.7 (0.4‐1.2)	0.8 (0.4‐1.1)	0.7 (0.4‐1.3)	0.372
Monocytes (×10^9^/L)	0.4 (0.2‐0.6)	0.4 (0.2‐0.6)	0.4 (0.3‐0.6)	0.723
PCT (μg/L)	0.3 (0.2‐5.5)	0.3 (0.1‐3.0)	0.4 (0.2‐6.3)	0.360
CRP (mg/L)	49.6 (24.5‐71.0)	53.4 (45.3‐80.6)	30.2 (14.7‐68.3)	0.129
HCT (l/L)	33.9 ± 11.2	33.6 ± 6.3	34.3 ± 14.7	0.826
ESR (mm/h)	67.0 (31.0‐82.0)	35.0 (20.5‐72.0)	73.0 (39.0‐112.0)	0.148
ALT (U/L)	36.0 (19.0‐69.0)	36.0 (17.5‐64.0)	34.5 (19.8‐75.0)	0.449
AST (U/L)	34.1 (20.0‐71.0)	32.0 (20.5‐84.0)	34.5 (20.0‐71.0)	0.442
ALP (U/L)	87 (67.7‐135.5)	93.0 (67.8‐105.0)	86.5 (68.0‐137.8)	0.622
GGT (U/L)	54.5 (31.0‐118.0)	47.0 (21.0‐84.0)	73.0 (34.0‐167.3)	0.324
TBIL (μmol/L)	20.7 (8.9‐41.0)	14.0 (8.2‐41.8)	24.9 (12.7‐41.3)	0.983
DBIL (μmol/L)	7.7 (4.0‐28.6)	5.4 (2.7‐27.4)	9.5 (5.6‐29.0)	0.673
Cr (μmol/L)	62 (48.0‐111.3)	55.5 (40.3‐78.8)	73.0 (55.8‐134.5)	0.508
Lactate (mmol/L)	2.1 (1.5‐3.2)	1.75 (1.2‐2.1)	2.6 (1.6‐3.5)	**0.039**
PH	7.3 ± 0.36	7.3 ± 0.47	7.4 ± 0.10	0.452
PaCO_2_ (mmHg)	36.9 ± 13.1	38.3 ± 15.3	35.6 ± 11.1	0.662
PaO_2_ (mmHg)	74.9 ± 23.5	84.2 ± 22.3	65.7 ± 21.6	**0.043**
Na^+^ (mmol/L)	135.8 ± 5.3	135.7 ± 4.8	136.0 ± 5.8	0.844
K^+^ (mmol/L)	3.8 ± 0.7	3.8 ± 0.7	3.8 ± 0.8	0.984

Abbreviations: PCT, procalcitonin; CRP, C‐reactive protein; HCT, red blood cell specific volume; ESR, erythrocyte sedimentation rate; ALT, alanine transaminase; AST, aspartate transaminase; ALP, alkaline phosphatase; GGT, gamma‐glutamyl transpeptidase; TBIL, total bilirubin; DBIL, direct bilirubin; Cr, creatinine.

### 2.2 Experimental models

Male adult C57BL/6J mice (20‐26 g) were purchased from the Experimental Animal Center of Xi'an Jiaotong University. Animals were anesthetized with isoflurane. Cecal ligation and puncture (CLP) induced sepsis was established with a 22‐gauge needle accordingly.^30^ At 5 h after CLP, normal saline (0.5 mL, vehicle) or irisin (0.5 mL, 250 μg/kg, 067‐29A, Phoenix Pharmaceuticals, USA) was administered intravenously. 30 mg/kg RSL3 (S8155, Selleck Chemicals, USA) was administrated intraperitoneally after irisin injection immediately. In addition, 15 or 45 mg/kg BW lipopolysaccharides (LPS) (*Escherichia coli* 055: B5, Sigma‐Aldrich) were administered intraperitoneally to establish a mice model of endotoxemia. The control group was injected with an equal volume of normal saline. All animals were treated with the Health Science Center (HSC) of Xi'an Jiaotong University criteria for use and care of animals in research. The experiments were approved by the Institutional Animal Care and Use Committee of the Ethics Committee of HSC.

**STUDY HIGHLIGHTS**

**What is the current knowledge on the topic?**

Sepsis remains a major health issue without an effective therapy. Ferroptosis has been proposed to be involved in the pathogenesis of sepsis. Irisin improves mitochondrial function under various conditions. Ferroptosis is closely related to mitochondrial function. However, the role of irisin in sepsis‐induced ferroptosis and mitochondrial dysfunction in the liver remained unknown.

**What question did this study address?**

We performed this study to assess the expression of irisin in sepsis, and investigate the effects of irisin on sepsis‐induced ferroptosis and mitochondrial dysfunction in the liver.

**What does this study add to our knowledge?**

Our results showed that serum irisin levels were decreased and negatively correlated with the APACHE II scores in patients with sepsis. Exogenous irisin administration can attenuate liver dysfunction, increase the expression of GPX4, which is down‐regulated in experimentally induced sepsis, and inhibit ferroptosis in sepsis.

**How might this change clinical pharmacology or translational science?**

Irisin may be a potential therapeutic agent for sepsis.

### 2.3 Cell lines and culture

The L‐02 hepatocyte line (abbreviated as L02 cells) was purchased from Procell Co., Ltd (Wuhan, China). Cells were cultured in RPMI‐1640 medium supplemented with 10% fetal bovine serum, 100 μg/mL streptomycin and 100 U/mL penicillin, and maintained in a humidified atmosphere with 5% CO_2_ at 37°C.

### 2.4 Enzyme‐linked immunosorbent assays

Irisin commercial ELISA kits (EK‐067‐29, Phoenix Pharmaceuticals, USA), TNF‐α ELISA kit (CSB‐E04741m, Wuhan, China) and IL‐6 ELISA kit (CSB‐E04639m, Wuhan, China) were used to measure the serum irisin, TNF‐α, and IL‐6 following the instructions.

### 2.5 Malondialdehyde, glutathione, and iron assays

Lipid Peroxidation (MDA) Assay Kit (S0131S, Beyotime, China), total Glutathione Assay Kit (S0052, Beyotime, China), and Iron assay kit (ab83366, Abcam, UK) were applied to assess the lipid peroxidation product MDA contents, GSH levels, and ferrous iron (Fe^2+^) concentrations following the instructions.

### 2.6 RNA extraction and quantitative real‐time RT‐PCR

The total RNA was extracted with TRIzol and the qPCR was carried out as described before.^31^ The specific primers for mouse FNDC5 were 5′‐GTC ATT GGC TTT GCC ATC TC‐3′ (forward) and 5′‐CAT GGA CGA TAT ATT CTG TGT CCT C‐3′ (reverse). The specific primers for mouse IL‐6, TNF‐α, IL‐1β, CXCL‐1, CXCL‐10, and β‐actin were the same as listed in the recent study.^32^

### 2.7 Analysis of mitochondrial DNA content

Total DNA was isolated with a commercial kit (69504, Qiagen, Valencia, CA) following the instructions. qPCR assay was applied to determine the mtDNA by amplifying mitochondrial‐encoded NADH dehydrogenase 1 (MT‐ND1). DNA fragments encoding MT‐ND1 were normalized with the nuclear‐encoded internal control of OCT4. The detailed experimental scheme is the same as before.^16^

### 2.8 Liver injury and ATP content

Serum ALT and AST were detected with kits (C009‐2 and C010‐2) from NanJing JianCheng Bioengineering Institute (Nanjing, China). ATP was detected using the ATP Assay Kit (S0026, Beyotime, China), and the ATP content was normalized to total protein with a unit of nmol per milligram protein (nmol/mg protein).

### 2.9 DHE and MitoTracker Red fluorescent staining

The 5 μm cryosections from liver were immediately stained with 5 μM dihydroethidium (DHE) (D7008, Sigma‐Aldrich) or 100 nM MitoTracker Red CMXRos (MTR) (M7512, Thermo Fisher Scientific) at 37°C for 30 min in a light‐protected incubator. After being rinsed with PBS for three times, slides were coverslipped without fixation. Similarly, LO2 cells were incubated with staining solution containing 5 μM DHE or 200 nM MTR. The samples were immediately imaged under a fluorescence microscope. The excitation/emission wavelengths for DHE and MTR were 490/610 nm and 579/599 nm, respectively.

### 2.10 Western blot analysis

Protein extraction and western blot analysis were conducted as described before.^31^ The primary antibodies included: anti‐TFAM antibody (ab131607, 1:1000), anti‐ATPB antibody (ab14730, 1:5000), anti‐GPX4 antibody (ab125066, 1:3000) and anti‐HMGB1 antibody (ab79823, 1:10000) from Abcam (Cambridge, UK), anti‐BCL2 antibody (3498, 1:1000), anti‐LC3B antibody (3868, 1:1000) and anti‐P62 antibody (5114, 1:1000) from Cell Signaling Technology (Danvers, MA, USA), HRP‐conjugated mouse anti‐ACTB (KC‐5A08, 1:5000), and HRP‐conjugated mouse anti‐GAPDH (KC‐5G5, 1:5000) from Aksomics (Shanghai, China).

The HRP‐conjugated secondary antibodies involved anti‐mouse IgG (KC‐MM‐035, 1:5000) and anti‐rabbit IgG (KC‐RB‐035, 1:5000) from Aksomics (Shanghai, China). The protein bands were visualized with the ChemiDoc MP system (Bio‐Rad, CA). ImageJ software was applied to quantify the band intensity, and the results are presented as the ratio of the target protein to GAPDH or ATCB.

### 2.11 Transmission electron microscopy

Seventy nanometer sections were sliced from the liver samples and stained with uranyl acetate and lead citrate. Hepatic ultrastructure evaluations were performed using a transmission electron microscope (H‐7650, Hitachi, Japan).

### 2.12 Statistical analysis

All data were analyzed with IBM SPSS 24.0 software. Two‐tailed Student's *t*‐test and ordinary one‐way ANOVA were used for the difference analysis between two groups and more than two groups, separately. The correlation between irisin and the severity of the disease was determined by linear regression analysis using Pearson's test. *P* < .05 indicated a significant difference.

## 3 RESULTS

### 3.1 Serum irisin levels in patients with sepsis versus healthy control subjects

**FIGURE 1:**
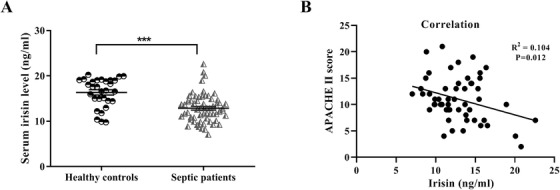
Serum irisin levels are decreased in patients with sepsis compared with healthy controls and negatively correlated with disease severity. A, Serum irisin levels in 60 septic patients and 29 normal individuals. Mean ± SEM, ^***^ *P* < .001. B, Correlation analysis of the Acute Physiology and Chronic Health Evaluation (APACHE) II score and serum irisin levels of septic patients. *P* = .012. The coefficient of correlation (*R* ^2^) and *P*‐value were determined by linear regression, and we considered *P* < .05 to indicate a correlation

Sixty patients with sepsis and 29 healthy subjects were involved in this study. As shown in Figure 1A, serum irisin levels in healthy subjects (16.35 ± 0.58 ng/mL) were notably higher than that in septic patients (12.85 ±0.41 ng/mL). The median level of serum irisin in septic patients was 12.67 ng/mL. Using this value as a cut‐off, patients with sepsis were divided into two groups. Table 1 showed that decreased serum irisin levels were related to history of viral hepatitis and cirrhosis, as well as a high lactate level and a low PaO_2_ level (*P* < .05 for all). In addition, a correlation was discovered between serum irisin levels in patients with sepsis and the APACHE II score, which is always used to determine disease severity of critical patients. In patients with sepsis, decreased serum irisin levels were inversely correlated with disease severity (*P* < .05; Figure 1B).

### 3.2 Serum irisin levels in septic animal models

**FIGURE 2:**
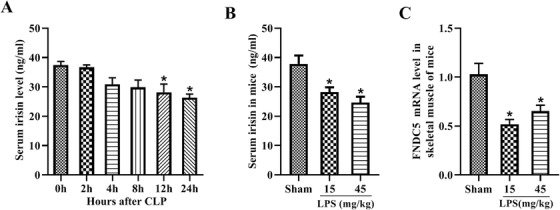
Serum irisin levels are decreased in experimentally induced sepsis. A, Serum irisin levels in CLP‐induced septic mice at 0, 2, 4, 8, 12, and 24 h after CLP surgery. n = 6, mean ± SEM, ^*^ *P* < .05 versus 0 h group. B, Serum irisin levels in LPS‐induced septic mice. n = 6, mean ± SEM, ^*^ *P* < .05 versus sham group. C, mRNA level of irisin in the skeletal muscle of LPS‐induced septic mice. n = 6, mean ± SEM, ^*^ *P* < .05 versus sham group

CLP and LPS‐induced septic animal models were established to verify the expression of irisin. Consistent with the clinical results, serum irisin was markedly reduced in CLP mice at 12 and 24 h postoperatively compared with the 0 h group (Figure 2A). The serum irisin levels were also reduced notably in the LPS‐induced mice and showed an LPS dose‐dependent decrease (Figure 2B). Irisin is mainly expressed in skeletal muscle, the mRNA level of FNDC5 in the skeletal muscle was notably decreased in LPS‐induced septic mice (Figure 2C), implicating a reduction of irisin in the skeletal muscle of septic mice.

### 3.3 Irisin suppressed ferroptosis in CLP‐induced septic mice

**FIGURE 3:**
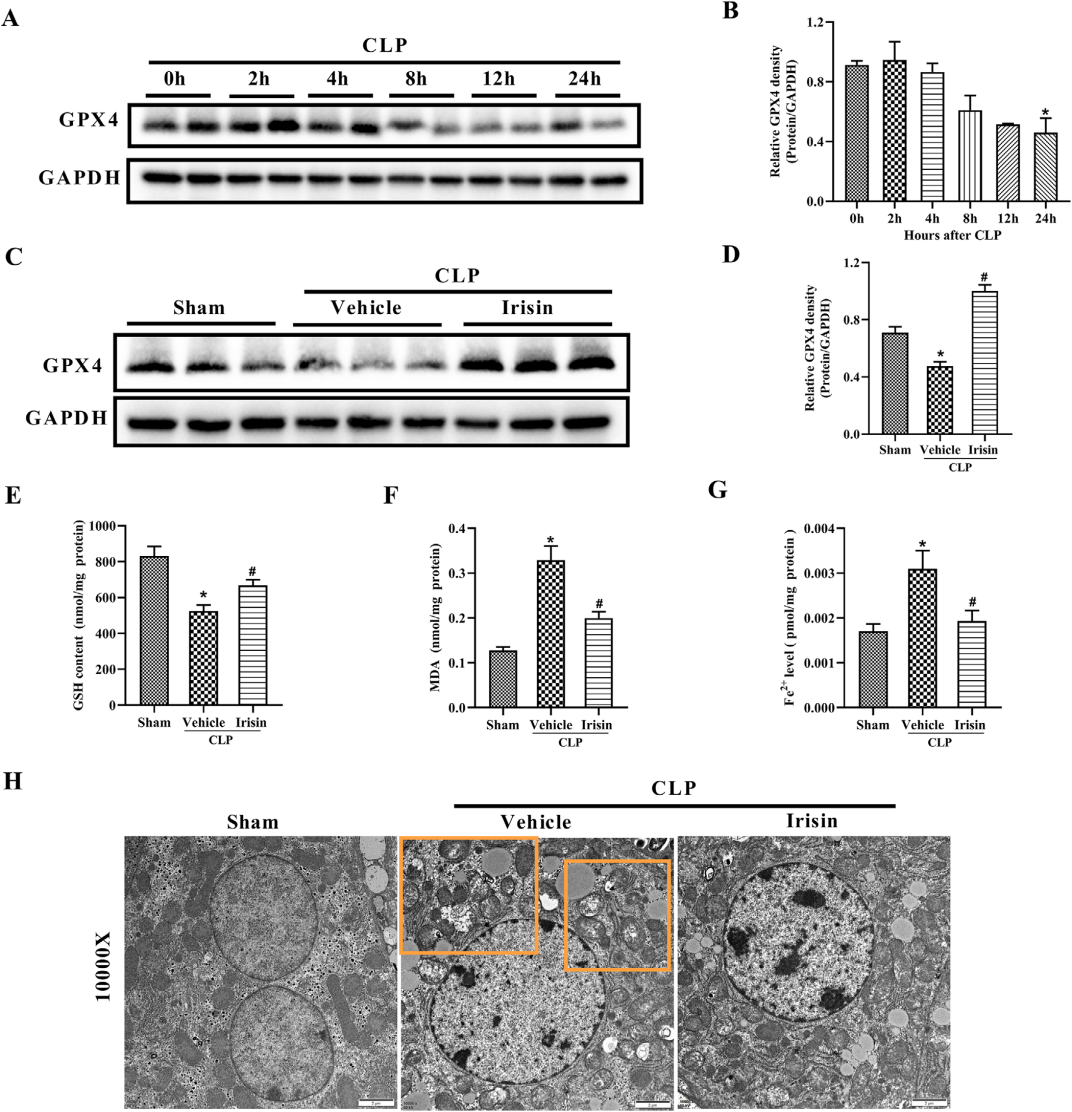
Irisin suppressed ferroptosis in CLP‐induced septic mice. A and B, Western blot analysis of GPX4 in the liver of CLP‐induced septic mice at 0, 2, 4, 8, 12, and 24 h after CLP surgery. n = 3, mean ± SEM, ^*^ *P* < .05 versus 0 h group. C and D, Western blot analysis of GPX4 in the liver of CLP‐induced septic mice 24 h after CLP surgery with irisin treatment. n = 3, mean ± SEM, ^*^ *P* < .05 versus sham group; **^#^** *P* < .05 versus CLP group. E, Liver glutathione (GSH) contents. F, Liver malondialdehyde (MDA) contents. G, Liver ferrous (Fe^2+^) concentrations. H, Overview of the ultrastructure of liver mitochondria by transmission electron microscopy (TEM). n = 6, mean ± SEM, ^*^ *P* < .05 versus sham group; **^#^** *P* < .05 versus CLP group

In order to evaluate ferroptosis in sepsis, glutathione peroxidase 4 (GPX4), which was essential to maintain lipid homeostasis in the cells and negatively associated with the levels of ferroptosis,^21^ was used to detect ferroptosis in septic mice. Figures 3A and 3B showed that GPX4 was obviously reduced in the liver of CLP mice at 24 h postoperatively compared with the 0 h group. Furthermore, GPX4 was also notably decreased in the liver of mice injected with 45 mg/kg LPS (Figure S2). However, irisin treatment obviously increased GPX4 expression in the liver of CLP mice (Figure 3C,D). In addition to the detection of GPX4 expression, GSH content, lipid peroxidation, iron accumulation, and mitochondrial morphology were critical evidence to characterize ferroptosis,^20^, ^33^ thus we investigated these biomarkers in septic mice. The data displayed that GSH contents were significantly decreased, MDA levels and Fe^2+^ levels were notably increased in the liver of septic mice, accompanied with mitochondrial shrinkage and increased bilayer density. However, irisin treatment reversed levels of these indicators and alleviated the mitochondrial impair in morphology (Figure 3E‐H). These results indicated that ferroptosis was enhanced in the liver of septic mice and irisin treatment decreased ferroptosis in septic mice. Besides liver, irisin also suppressed ferroptosis in the lung of CLP mice (Figure S3).

### 3.4 Irisin inhibits inflammatory response in CLP‐induced septic mice

**FIGURE 4:**
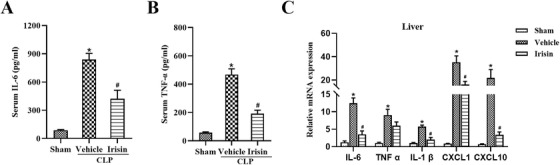
Irisin administration reduces levels of inflammatory factors in the serum and liver tissues of CLP‐ induced septic mice. A and B, Serum levels of inflammatory factors (IL‐6, TNF‐α) in septic mice. C, Relative mRNA expression levels of inflammatory factors (IL‐6, TNF‐α, IL‐1β, CXCL1, and CXCL10) in the liver of septic mice after normalization with ATCB mRNA levels as determined by qRT‐PCR. n = 6, mean ± SEM, ^*^ *P* < .05 versus sham group; ^#^ *P* < .05 versus CLP group

Excessive increase of inflammatory factors led to organ damage in sepsis. To confirm whether irisin could reduce inflammatory response in sepsis, the expression levels of inflammatory factors in serum and vital organs were detected. It was demonstrated that irisin administration markedly reduced serum IL‐6 and TNF‐α levels in septic mice (Figure 4A,B). Relative mRNA expression levels of IL‐6, IL‐1β, CXCL1, and CXCL10 in the liver of septic mice were significantly decreased with irisin treatment (Figure 4C). Similarly, notable reduction of IL‐6 and IL‐1β in kidney, IL‐6, TNF‐α, and IL‐1β in lung, IL‐6, TNF‐α, and CXCL1 in heart were observed in irisin‐treated septic mice (Figure S5). Taken together, irisin inhibits systemic inflammation in septic mice.

### 3.5 Irisin administration attenuates mitochondrial damage in septic mice

**FIGURE 5:**
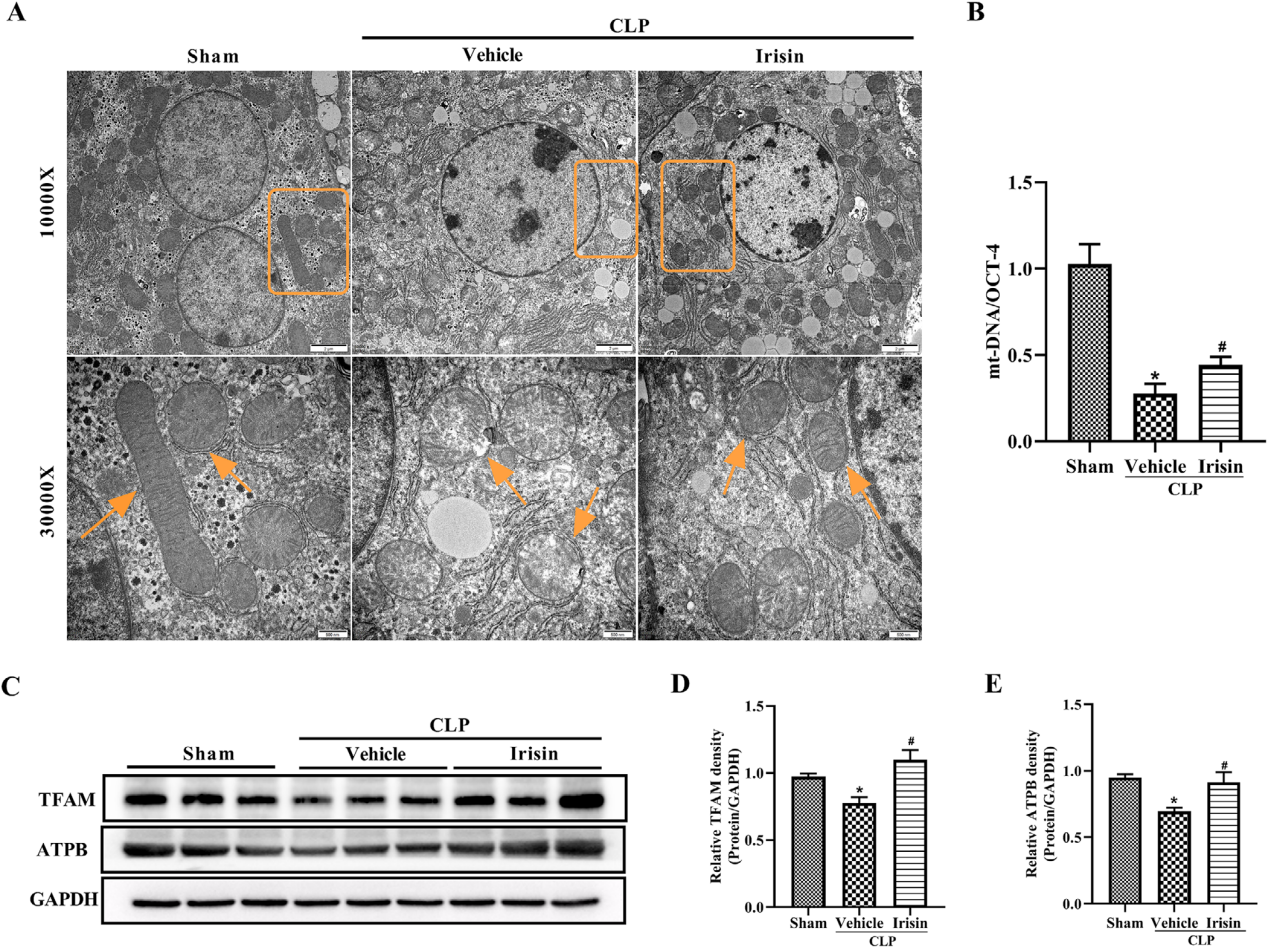
Irisin administration attenuates mitochondrial damage in septic mice. Irisin treatment in mice was conducted by intravenous administration (250 μg/kg, a single dose) 5 h after CLP surgery. Liver tissues were harvested 24 h after CLP surgery. A, Overview of the ultrastructure of liver mitochondria by transmission electron microscopy (TEM), images for the sham group were the same as that in Figure 3. B, Liver mtDNA copy numbers as assessed by quantitative polymerase chain reaction (qPCR). n = 6, mean ± SEM, ^*^ *P* < .05 versus sham group; **^#^** *P* < .05 versus CLP group. C‐E, Western blot analysis of TFAM and ATPB expression in the liver. n = 6, mean ± SEM, ^*^ *P* < .05 versus sham group; **^#^** *P* < .05 versus CLP group

Serum ALT and AST were moderately increased in CLP mice at 24 h postoperatively and notably reduced by irisin administration (*P* < .05; Figure S1). The structure of liver tissue was observed by transmission electron microscopy (TEM). As shown in Figure 5A, compared with the sham group, CLP operation induced mitochondrial swelling, disappearance of mitochondrial cristae, rupture of the outer mitochondrial membrane (OMM), and swelling of the endoplasmic reticulum. These alterations were significantly alleviated in the irisin treatment group. To explore whether irisin had an influence on mitochondrial biogenesis, mtDNA copy number (mtDNA/nDNA) was detected by qPCR and it was markedly decreased in the vehicle group, while exogenous irisin treatment increased the mtDNA copy number (Figure 5B).

Mitochondrial swelling and OMM rupture imply damage of mitochondrial function, thus we detected the expression of ATPB (energy generation related protein) and TFAM (mitochondrial biogenesis related protein). Similar to the change in mtDNA copy number, western blotting indicated that both these proteins were markedly downregulated in the CLP group, while they were notably upregulated in the irisin‐treated group (Figure 5C‐E).

### 3.6 Irisin administration increases mitochondrial activity and ATP production in septic mice and LPS‐treated L02 cells

**FIGURE 6:**
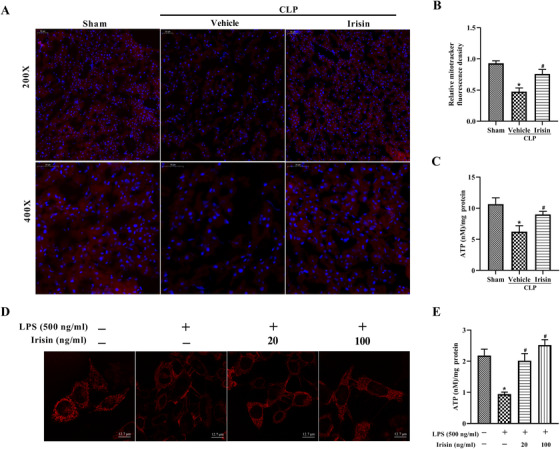
Irisin administration increases mitochondrial activity and ATP production in septic mice and LPS‐treated L02 cells. Irisin treatment in mice was conducted by intravenous administration (250 μg/kg, a single dose) 5 h after CLP surgery. Liver tissues were harvested 24 h after CLP surgery. L02 cells were treated with 20 or 100 ng/mL irisin in the presence of 500 ng/mL LPS for 24 h. A and B, MitoTracker Red CMXRos fluorescence staining and the fluorescence intensity of liver tissues. C, ATP content of liver tissues. D, MitoTracker Red CMXRos fluorescence staining of L02 cells. E, ATP content of L02 cells. For septic mice, n = 6, mean ± SEM, ^*^ *P* < .05 versus sham group; **^#^** *P* < .05 versus CLP group. For L02 cells, n = 3, mean ± SEM, ^*^ *P* < .05 versus blank group; **^#^** *P* < .05 versus LPS group

Mitochondrial activity in the liver was evaluated by MitoTracker Red, and the red fluorescence intensity was markedly decreased in CLP‐induced septic mice. However, treatment with exogenous irisin notably increased the fluorescence intensity (Figure 6A,B). Moreover, the liver ATP content could reflect mitochondrial energy metabolism. ATP content was reduced dramatically in the vehicle group, while irisin treatment markedly increased the ATP content (Figure 6C). The treatment of L02 cells with LPS for 24 h was designed to mimic septic infection in the liver. The fluorescence intensity of MitoTracker Red and ATP content were significantly decreased in the LPS‐treated L02 cells, while irisin treatment could reverse this effect (Figure 6D,E).

### 3.7 Irisin administration reduces oxidative stress in septic mice and LPS‐treated L02 cells

**FIGURE 7:**
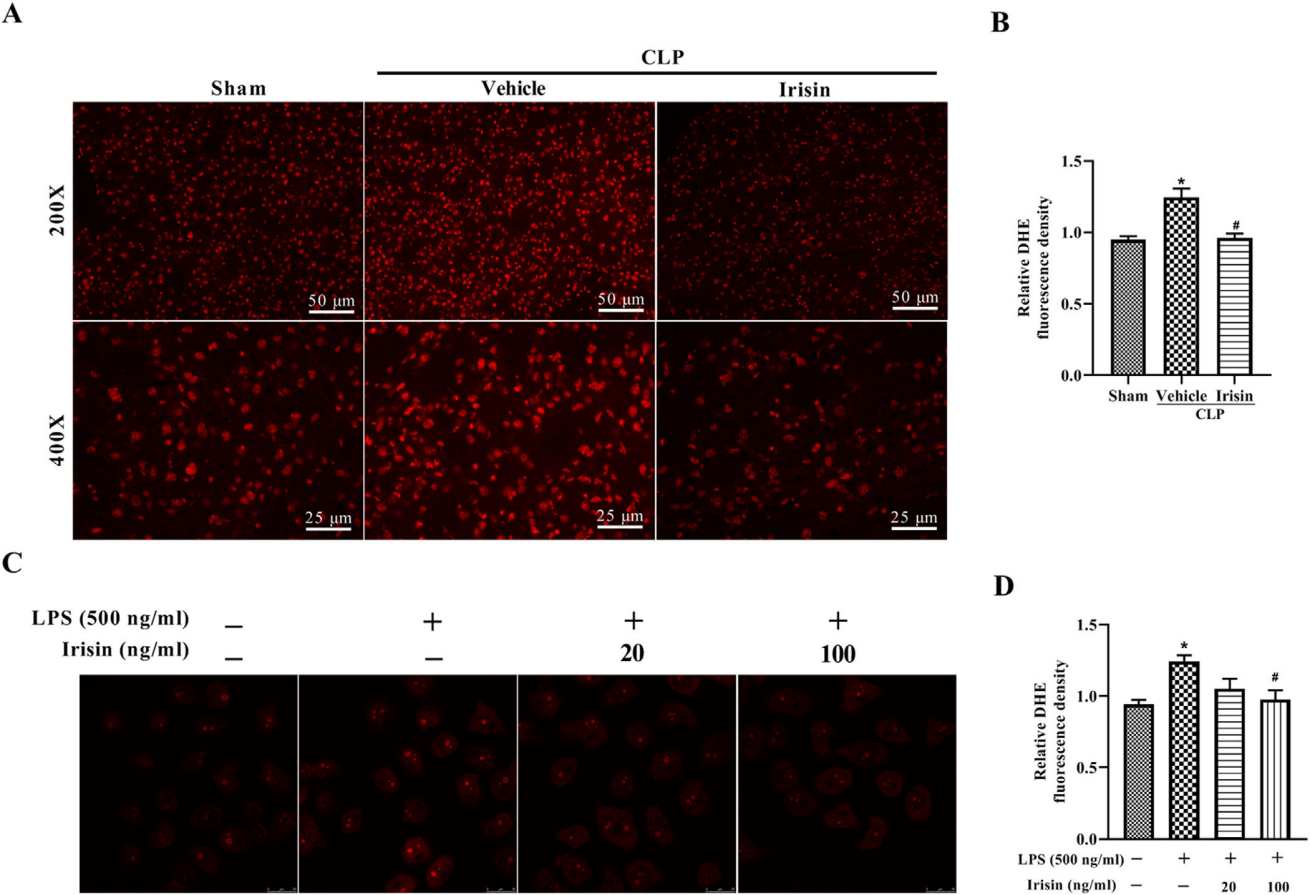
Irisin administration reduces oxidative stress in septic mice and LPS‐treated L02 cells. A and B, DHE fluorescence staining and fluorescence intensity in liver tissues. C and D, DHE fluorescence staining and fluorescence intensity in L02 cells. For septic mice, n = 6, mean ± SEM, ^*^ *P* < .05 versus sham group; **^#^** *P* < .05 versus CLP group. For L02 cells, n = 3, mean ± SEM, ^*^ *P* < .05 versus blank group; **^#^** *P* < .05 versus LPS group

DHE staining was conducted to evaluate tissue ROS after CLP operation for 24 h with and without the supplement of exogenous irisin. The results showed significantly increased ROS in the vehicle group, but irisin treatment greatly reduced the production of ROS (Figure 7A,B). A similar situation regarding ROS level was observed the LPS‐treated L02 cells, and 100 ng/mL irisin treatment could reverse this effect (Figure 7C,D). In conclusion, irisin treatment regulated oxidative stress in sepsis.

### 3.8 GPX4 inhibitor eliminates irisin's effects in sepsis

**FIGURE 8:**
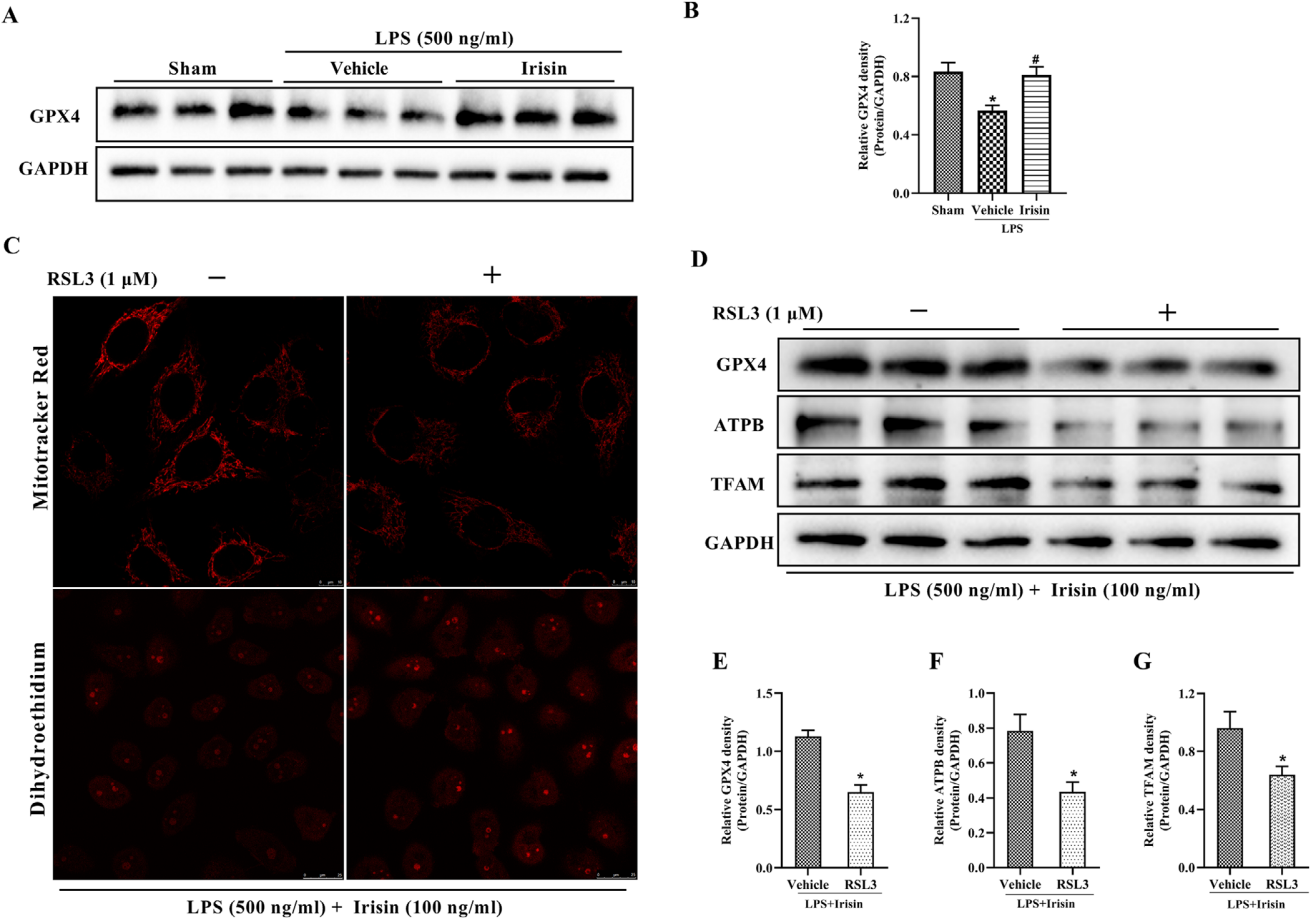
RLS3 is a GPX4 inhibitor to induce ferroptosis and can eliminate irisin's effects in LPS‐treated L02 cells. A and B, Western blot analysis of GPX4 in irisin‐treated L02 cells in the presence of LPS. n = 3, mean ± SEM, ^*^ *P* < .05 versus sham group; ^#^ *P* < .05 versus LPS group. C, MitoTracker Red CMXRos fluorescence staining and DHE fluorescence of L02 cells treated with RLS3 in the presence of 100 ng/mL irisin and 500 ng/mL LPS for 24 h. D‐G, Western blot analysis of GPX4, TFAM, and ATPB expression in L02 cells treated with RLS3 in the presence of 100 ng/mL irisin and 500 ng/mL LPS for 24 h. n = 3, mean ± SEM, ^*^ *P* < .05 versus LPS+ irisin group

**FIGURE 9:**
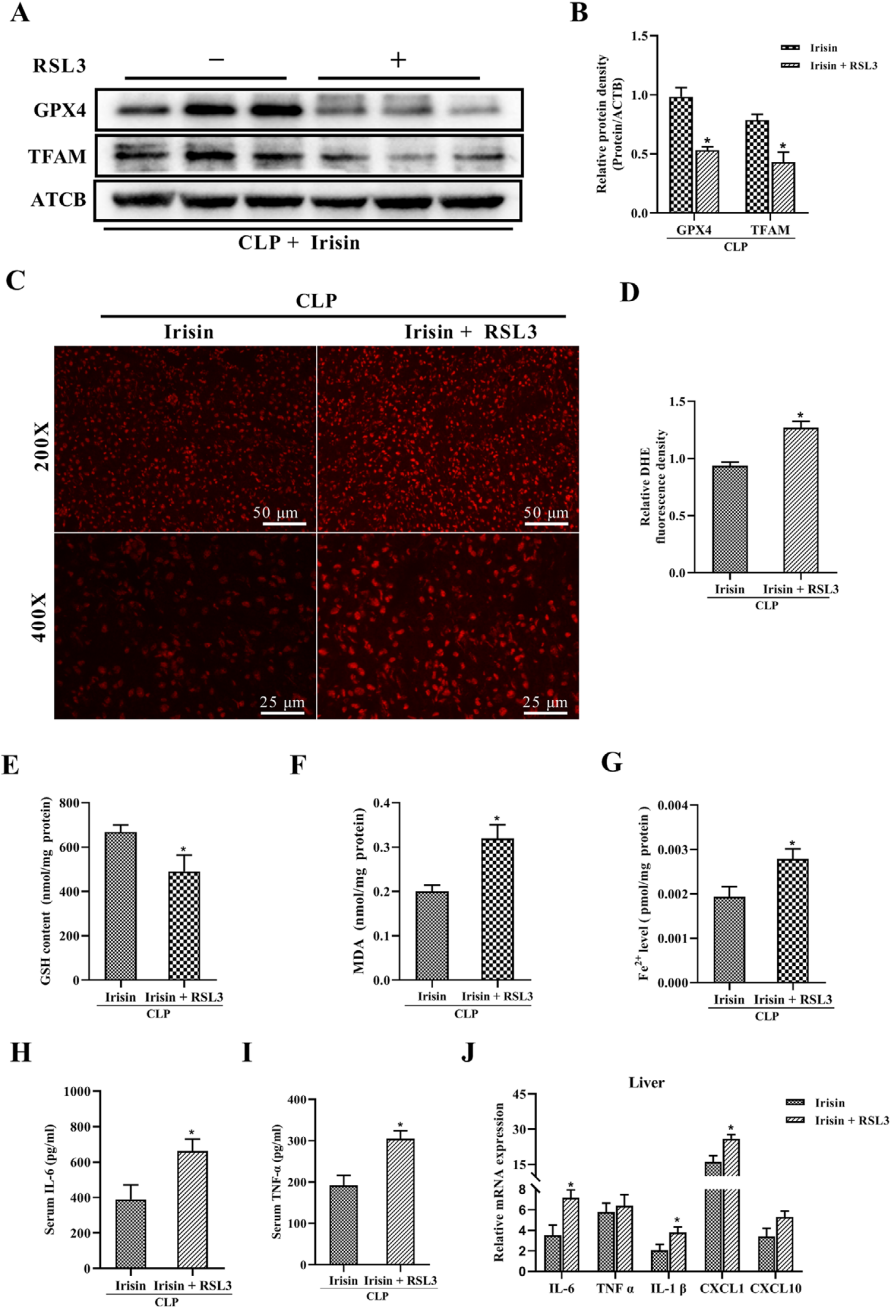
RLS3 eliminated irisin's effects in CLP‐mice by promoting ferroptosis. A and B, Western blot analysis of GPX4 and TFAM in the liver of septic mice. C and D, DHE fluorescence staining and fluorescence intensity in liver tissues. E, Liver glutathione (GSH) contents. F, Liver malondialdehyde (MDA) contents. G, Liver ferrous (Fe^2+^) concentrations. H and I, Serum cytokine concentrations (IL‐6, TNF‐α) in septic mice. J, Relative mRNA expression levels of cytokines (IL‐6, TNF‐α, IL‐1β, CXCL1, and CXCL10) in the liver of septic mice after normalization with ATCB mRNA levels as determined by qRT‐PCR. n = 6, mean ± SEM, ^*^ *P* < .05 versus irisin group

L02 cells were applied to investigate whether ferroptosis participates in the protective effects of irisin in sepsis. A remarkable downregulation of GPX4 was observed in LPS‐treated L02 cells, which was consistent with our in vivo experimental results, while irisin treatment significantly increased the protein level of GPX4 (Figure 8A,B). RSL3, a GPX4‐specific inhibitor to induce ferroptosis,^34^ was applied to detect mitochondrial activity and ROS production in vitro. After being treated with RSL3, LPS and irisin for 24 h, the fluorescence intensity of MitoTracker Red in L02 cells was significantly decreased and the production of ROS was greatly increased (Figure 8C). Meanwhile, the protein expression of GPX4, ATPB, and TFAM were notably downregulated in L02 cells treated with RSL3 (Figure 8D‐G). Downregulation of GPX4 showed a strong inhibition effect of RSL3, and downregulation of ATPB and TFAM indicated impaired mitochondrial function after RSL3 treatment. Furthermore, RSL3 was administered together with irisin in mice at 5 h after CLP surgery. The protein levels of GPX4 and TFAM were notably reduced (Figure 9A,B), while ROS generation was greatly elevated in the RSL3‐treated group (Figure 9C,D), which were consistent with the results in vitro. In addition, RSL3 treatment abolished the beneficial effects of irisin on GSH, MDA, and Fe^2+^ levels (Figure 9E‐G). The serum levels of ALT, AST (Figure S1), IL‐6, and TNF‐α (Figure 9H,I), and the mRNA expression of IL‐6, IL‐1β, and CXCL‐1 in liver were notably elevated with RSL3 treatment (Figure 9J). The results above exhibited that RSL3 impaired the protective effects of irisin on liver mitochondrial function, ROS production, liver injury, and systemic inflammation via suppression of GPX4. In conclusion, enhanced ferroptosis eliminated the protective effects of irisin in sepsis.

### 3.9 Integrin inhibitors eliminate irisin's effects on GPX4 and mitochondrial function

**FIGURE 10:**
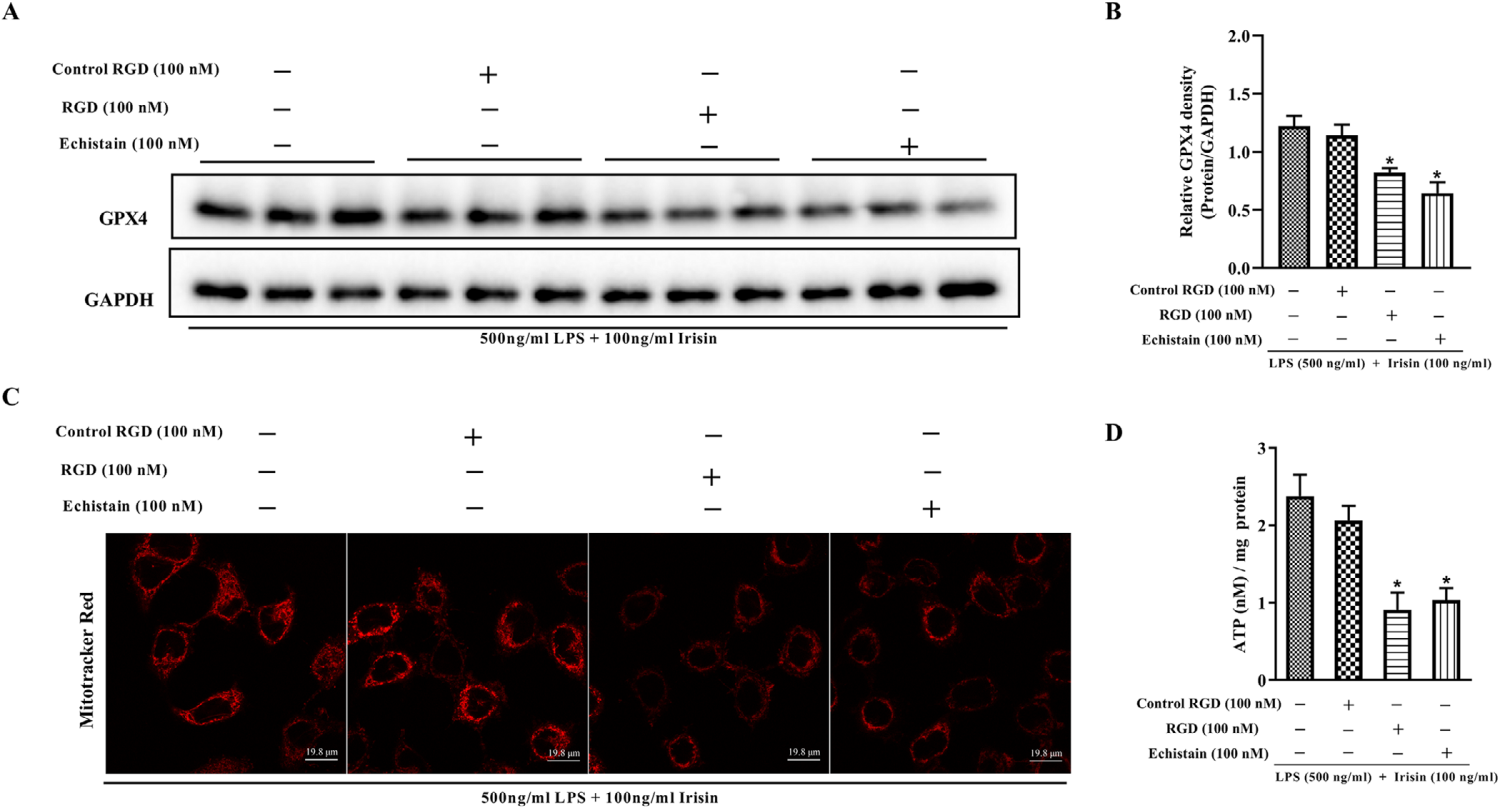
Integrin inhibitors eliminate irisin's effects on GPX4 and mitochondrial function. aV integrins was identified as the receptor of irisin, L02 cells were treated with integrin‐inhibitor (RGD peptide, Echistain) in the presence of 100 ng/mL irisin and 500 ng/mL LPS for 24 h. A and B, Western blot analysis of GPX4 in L02 cells treated with integrin inhibitor. C, MitoTracker Red CMXRos fluorescence staining of L02 cells treated with integrin inhibitor. D, ATP content of L02 cells treated with integrin inhibitor. n = 3, mean ± SEM, ^*^ *P* < .05 versus blank group

Recently, αV integrins have been identified as the receptor of irisin that stimulate signal transduction in an osteocyte cell line.^35^ To determine whether irisin could affect ferroptosis and hepatic mitochondria, inhibitors of the irisin receptor (RGD peptide and Echistain) were used to treat L02 cells in the presence LPS and irisin for 24 h. Compared with the irisin group and the negative control RGD group, the protein expression of GPX4, MitoTracker fluorescence intensity, and the ATP content were significantly decreased in both of the integrin inhibitor‐treated groups (Figure 10). The inhibition of irisin's effects promoted ferroptosis and impaired mitochondrial function in the irisin and LPS‐treated L02 cells.

## 4 DISCUSSION

As a new member of muscle‐derived myokines discovered in 2012, irisin has received much attention because of its relevance to the browning of adipocytes and thermogenesis. It has emerged as an appealing therapeutic target for metabolic diseases. Most of studies showed that serum irisin levels were decreased in patients with type 2 diabetes mellitus (T2DM), nonalcoholic fatty liver disease (NAFLD), cardiovascular disease and metabolic bone disease,^36^, ^37^ as well as in infants born with neonatal respiratory distress syndrome (NRDS).^38^ However, the level of irisin in the serum of patients with sepsis has not been reported. In this study, we first found that serum levels of irisin were decreased in patients with sepsis and in animal models of sepsis induced by CLP and LPS. These results suggest that there might be an irisin deficiency in sepsis. The liver is a mitochondria‐rich organ since most of its functions are dependent on the energy primarily generated by mitochondria.^39^ Mitochondria dysfunction results in reduced ATP content and elevated ROS levels.^40^ During sepsis, mitochondrial ROS production directly leads to mitochondrial damage, which results in a ROS‐induced ROS release phenomenon and further mitochondrial damage, finally leading to mitochondrial dysfunction and subsequent bioenergetic failure in organs.^10^, ^41^ Mitochondrial oxidative stress is an important factor causing organ dysfunction in sepsis.^42^ Clinical research has shown that total serum antioxidant capacity levels reflect the clinical severity of sepsis, suggesting damage to oxidative stress (OXS) in patients with sepsis.^43^ The acute impairment of hepatic mitochondrial function was discovered in an animal model of sepsis with ultrastructural changes, considered a sign of mitochondrial dysfunction.^44^ Consistent with previous reports, our study also shows remarkable changes in mitochondrial morphology and a significant increase in ROS, as well as a remarkable decrease in ATP in the liver of septic mice, suggesting the occurrence of hepatic mitochondrial dysfunction.

Studies have confirmed that irisin alleviates damage under pathological conditions by protecting mitochondrial function, especially in ischemia/reperfusion injury,^16^, ^38^, ^45^, ^46^ implying that mitochondria are a target for irisin. It has been demonstrated that irisin could reduce oxidative stress under pathological conditions.^16^, ^17^, ^18^, ^19^, ^47^ Nevertheless, studies associated with the role of irisin in sepsis are scarce. A study on sepsis‐induced acute lung injury demonstrated that irisin ameliorated mitochondrial vacuolization, ridge dissolution, and emptied lamellar bodies in lung tissues of LPS‐induced septic rats.^48^ Tan et al reported that irisin corrected mitochondrial fission, increased the ATP production, and reduced mitochondrial ROS in septic cardiomyopathy of mice.^49^ Similarly, in the current study, irisin reversed abnormal mitochondrial morphology, reduced ROS production, and increased the ATP content in the liver of CLP mice and LPS‐treated hepatocytes. In an in vitro experiment, we verified the protective effect of irisin on sepsis by detecting mitochondrial activities using integrin inhibitors. These results confirmed the role of integrin in irisin's biological functions.

Mitochondrial biogenesis is critical for mitochondrial homeostasis and intracellular physiological demands; impaired mitochondrial biogenesis inhibits mtDNA transcription and contributes to mitochondrial dysfunction.^50^ Mitochondrial transcription factor A (TFAM), which controls the transcription and replication of mtDNA, is an important factor regulating mitochondrial biogenesis. Consistent with the decreased expression of TFAM in liver ischemia‐reperfusion injury and hippocampal tissues from AD brains,^16^, ^50^ our study also showed that the expression of TFAM in the liver was notably decreased in septic mice. In addition, the mtDNA copy number was decreased. These results indicate the reduced mitochondrial biogenesis in sepsis. However, the TFAM level and mtDNA copy number in the liver were increased by irisin treatment. Thus, we speculate that irisin might increase mtDNA copy number by upregulating the expression of TFAM in the liver.

Ferroptosis is caused by the elevation of iron levels and excessive accumulation of lipid ROS.^21^ Glutathione peroxidase 4 (GPX4) is a lipid repair enzyme that can catalyze glutathione (GSH) to oxidized glutathione disulfide (GSSG), eliminate lipid peroxides, and protect cell membrane against peroxidation of polyunsaturated fatty acids.^21^, ^51^ Yang et al demonstrated that the GPX4 is the key upstream regulator of ferroptosis in 2014.^34^ It has been reported that GPX4 activity decreases can lead to ferroptosis.^26^, ^52^ In our study, GPX4 was also notably reduced in the liver of CLP or LPS‐induced septic mice, the levels of ROS, MDA, and Fe^2+^ were obviously increased, while GSH contents were significantly decreased in the liver of CLP‐induced septic mice, suggesting activation of ferroptosis in the liver of septic mice. However, irisin treatment notably elevated GPX4 levels and GSH contents, markedly reduced MDA and Fe^2+^ levels in the liver of CLP‐mice. In addition, the level of GPX4 and irisin's effects on GPX4 were confirmed in LPS‐treated hepatocytes, which were in accordance with the conditions in septic mice. These in vivo and in vitro results indicated that irisin might exert the protective effects in sepsis‐induced liver dysfunction via inhibition of ferroptosis. We also measured GPX4 expression in the kidney, lung, and heart of CLP mice. The expression of GPX4 was only significantly decreased in the lung of septic mice. While irisin treatment notably enhanced the expression of GPX4 in the lung. Although GPX4 in the heart of septic mice was slightly reduced, the differences were not statistically significant. However, Wang et al reported that the expression of GPX4 was markedly decreased in the heart of CLP mice, accompanied with elevated iron concentration.^27^ This difference might due to the operation in animal model construction.

To investigate the role of ferroptosis in irisin's effects in sepsis, RSL3 was used to inhibit GPX4 and induce ferroptosis. It has been verified that RSL3 treatment increased the lipid ROS level and inhibited GPX4 in cultured cells,^34^ we also found that RLS3 treatment decreased GPX4 protein levels and GSH contents, promoted the generation of ROS and MDA, increased Fe^2+^ accumulation, and impaired mitochondrial activity and function by downregulation of TFAM and ATPB in the irisin and LPS‐treated hepatocytes. Consistently, RSL3 administration in CLP mice demonstrated that irisin indeed works on ferroptosis for sepsis protection. Furthermore, inhibitors of the irisin receptor (RGD peptide and Echistain) were used to confirm the regulation of irisin to ferroptosis in sepsis. It was observed that the expression of GPX4, mitochondrial activity, and the ATP content were significantly decreased in RGDS or Echistain‐treated hepatocytes in the presence of irisin and LPS. Taken together, irisin exerts the protective effects in sepsis‐induced liver dysfunction via suppressing ferroptosis.

Studies on septic animals have demonstrated that the expression of inflammatory factors were markedly elevated in serum and vital organs such as liver,^53^ lung,^48^ kidney,^54^ and heart.^49^ However, irisin treatment notably inhibited the inflammation injury in heart, lung, and kidney.^48^, ^49^, ^54^ Consistently with these previous results, we found that irisin administration markedly reduced serum IL‐6 and TNF‐α levels, as well as the mRNA expression levels of inflammatory factors in the liver, lung, kidney, and heart tissues of septic mice. The CLP model of sepsis in rodents does not usually lead to dramatic increases in serum levels of ALT and AST. Our findings are consistent with several other reports.^53^, ^55^, ^56^, ^57^ Lactate dehydrogenase (LDH) could be used together with ALT and AST to evaluate liver injury.^57^ In addition, BCL2 (biomarker of apoptosis),^58^ HMGB1 (biomarker of necrosis),^24^ LC3B, and P62 (biomarker of autophagy)^59^ were used to investigate whether irisin could regulate different types of cell death in liver during sepsis. The western blot results showed that irisin had an inhibitory effect on apoptosis in the liver of septic mice besides ferroptosis.

Irisin showed antioxidant, anti‐apoptotic, and anti‐inflammatory properties in recent studies.^48^, ^49^, ^54^, ^60^ Irisin alleviated mitochondrial dysfunction by improving mitochondrial membrane potential, sustaining mitochondrial respiration, decreasing mitochondrial ROS and MDA levels, suppressing DRP1‐related mitochondrial fission, and increasing cellular antioxidants in septic myocardial injury.^49^, ^60^ Studies have revealed that mitochondria were the physical interaction targets of irisin in heart and lung tissues with ischemia/reperfusion injury.^45^, ^61^ Irisin interacted with mitochondrial uncoupling protein 2 (UCP2) to reduce oxidative stress and restore mitochondrial function in lung injury.^61^ UCP2, a mitochondrial transporter protein uncoupling oxidative phosphorylation from ATP synthesis, was upregulated in the heart, liver, and other tissues of septic models, and showed a protective effect on mitochondrial injury in sepsis.^10^, ^62^, ^63^ Ferroptosis was characterized with accumulation of lipid peroxidation and iron overload, and was negatively regulated by GPX4.^21^, ^52^ Mitochondria were the center of oxidative metabolism and played an important role in ferroptosis,^28^ while lipid peroxidation and GPX4 were critical targets for treating sepsis.^26^ Taken together, the above findings indicated that mitochondria‐targeting irisin might inhibit the occurrence of ferroptosis in sepsis. Thus, we speculated that irisin alleviated mitochondrial dysfunction by downregulating UCP2, and showed the ability to suppress ferroptosis in septic mice via decreasing lipid peroxidation and increasing GPX4 expression.

The major strength of this study is that we investigated the role of irisin in both clinical and experimental sepsis. However, our study also has several limitations. First, the clinical cohort only consisted of samples from a single center and may not be generalizable to the rest of the country. Second, although this study has demonstrated the protective effects of irisin on the liver of septic models via suppressing ferroptosis, we did not address the precise mechanism related to the ferroptosis regulation. Further research is required to elucidate this mechanism. Furthermore, clinical studies should be conducted to validate the protective effect of irisin on sepsis‐associated ferroptosis and mitochondrial dysfunction.

## 5 CONCLUSIONS

Serum irisin levels are decreased and negatively correlated with disease severity in patients with sepsis, and irisin treatment suppresses ferroptosis and restores mitochondrial function in experimental sepsis. Irisin may be exploited as a new therapeutic option for patients with sepsis.

## ETHICS APPROVAL AND CONSENT TO PARTICIPATE

The study was approved by the Ethics Committee of the First Affiliated Hospital of Xi'an Jiaotong University, Shaanxi Province, China. All animal experiments were approved by the Institutional Animal Care and Use Committee of the Ethics Committee of Xi'an Jiaotong University Health Science Center, Shaanxi Province, China. All patients received a written informed consent before sample collection.

## CONSENT FOR PUBLICATION

Written informed consent for publication of this study was obtained from all participants.

## CONFLICT OF INTEREST

The authors have declared no conflict of interest.

## FUNDING

This work was supported by grants from the National Nature Science Foundation of China (No. 81701960 and 81770491) and the Ministry of Education Innovation Team Development Program of China (No. IRT16R57).

## AUTHOR CONTRIBUTIONS

SW designed and performed the experiments, analyzed the data, and wrote the manuscript. JB and LY performed the surgical procedure. JZ provided assistance in culturing cells. YW collected the samples. XC provided assistance in statistical analysis. YW and ZW collected clinical serum samples. YL supervised the study. RW designed the study and wrote the manuscript. All authors read and approved the final manuscript.

## Supporting information

SUPPORTING INFORMATION

## Data Availability

All data relevant to the study are included in the article or uploaded as Supporting Information.
