# Supplementary material for: Serum irisin levels are decreased in patients with sepsis, and exogenous irisin suppresses ferroptosis in the liver of septic mice
Source: Clin Transl Med. 2020 Sep 29;10(5):e173. doi: 10.1002/ctm2.173 (PMC7522760; doi:10.1002/ctm2.173)
Supplement: Supplementary file 1 — SUPPORTING INFORMATION [file CTM2-10-e173-s001.docx]

**Serum irisin levels are decreased in patients with sepsis, and** **exogenous irisin suppresses ferroptosis in the liver of septic mice**

**Running title: Irisin reduces sepsis-induced ferroptosis**

**Supplemental Files**

**
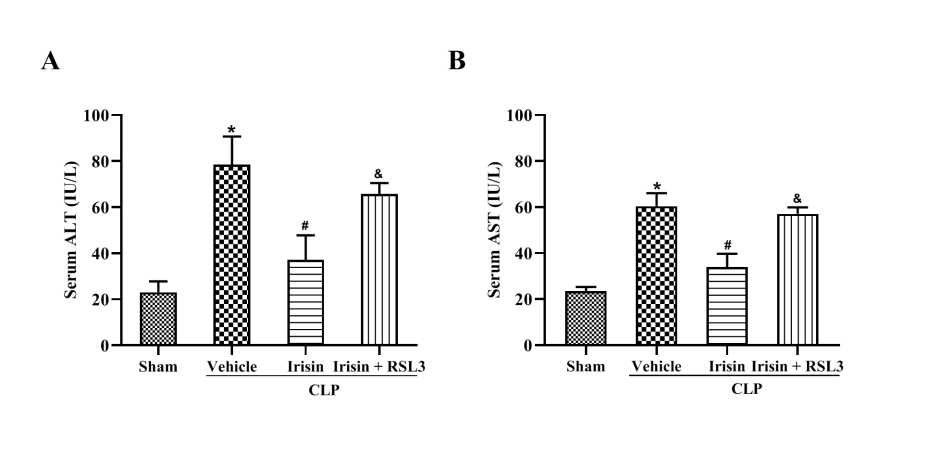
**

**Figure S1.** Effects of irisin and RSL3 administration on liver injury in mouse serum. The serum levels of alanine aminotransferase (ALT) and aspartate aminotransferase (AST) in sham , CLP-induced septic mice, and CLP-induced septic mice with irisin or irisin+RSL3 treatment. n = 6, mean ± SEM, *p < 0.05 vs. sham group; **^#^**p <0.05 vs. CLP group**;** ^&^p <0.05 vs. Irisin group.


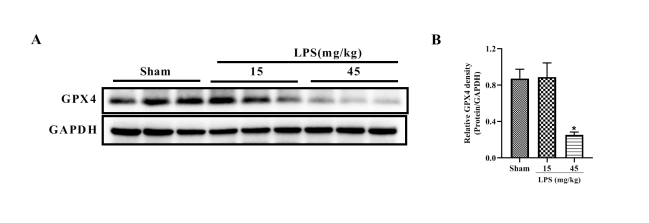


**Figure S2.** Ferroptosis was enhanced in LPS-induced septic mice. Western blot analysis of GPX4 in the liver of LPS-induced septic mice. n = 3, mean ± SEM, *p < 0.05 vs. sham group; **^#^**p <0.05 vs. LPS group.


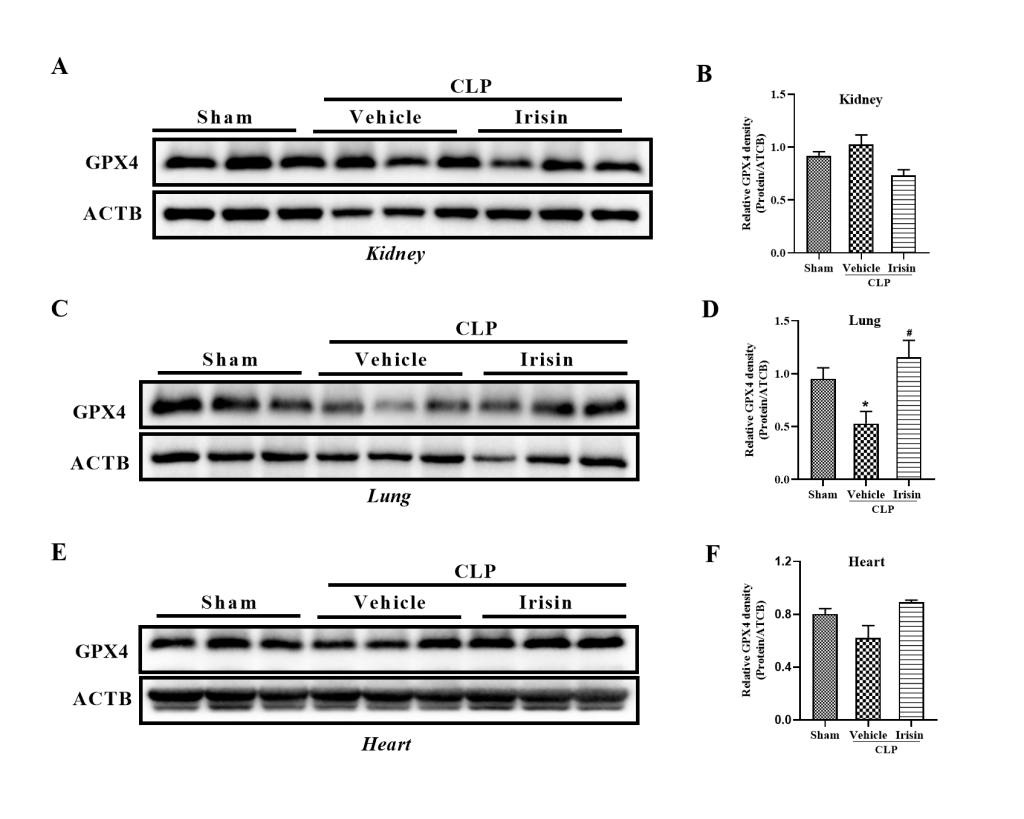


**Figure S3.** Effects of irisin on ferroptosis in the kidney, lung and heart tissues of CLP-induced septic mice. Changes in the protein expression levels of GPX4. n = 6, mean ± SEM, *p < 0.05 vs. sham group; **^#^**p <0.05 vs. CLP group.


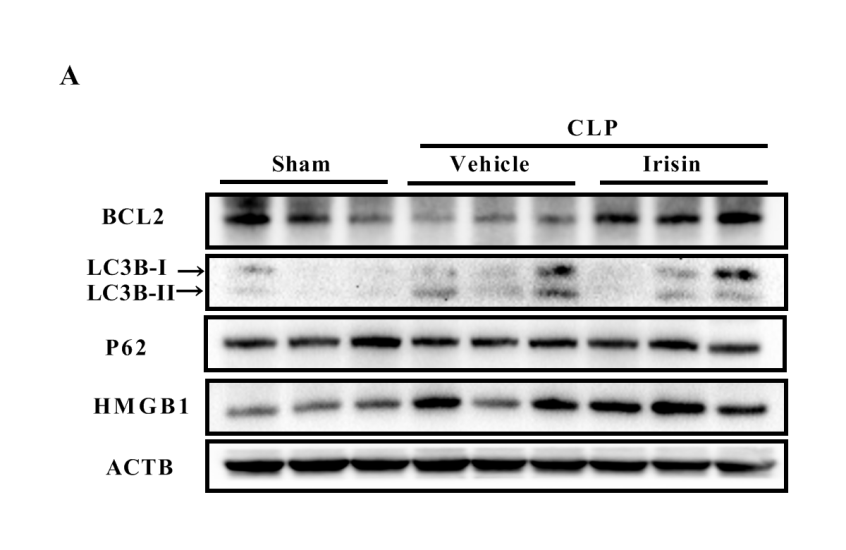


**Figure S4.** Effects of irisin on apoptosis, autophagy and necrosis during sepsis in the liver of CLP-induced septic mice. Changes in the protein expression levels of BCL2, LC3B, P62, HMGB1 as determined by western blot. n = 6, mean ± SEM, *p < 0.05 vs. sham group; **^#^**p <0.05 vs. CLP group.


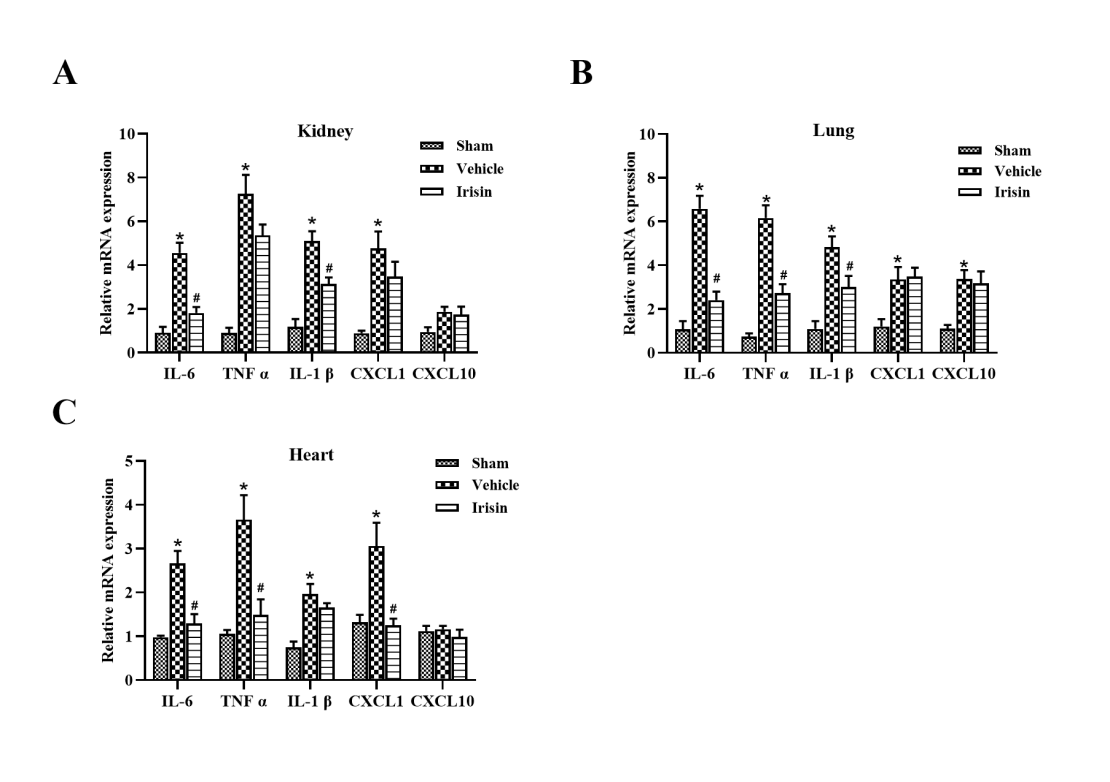


**Figure S5.** Irisin administration reduces inflammatory factors levels in the CLP-induced septic mice. Relative mRNA expression levels of inflammatory factors (IL-6, TNF-α, IL-1β, CXCL1 and CXCL10) in the kidney, lung and heart tissues. n = 6, mean ± SEM, *p < 0.05 vs. sham group; **^#^**p <0.05 vs. CLP group.
